# Strategic Priorities of the Scientific Plan of the European Research Infrastructure METROFOOD-RI for Promoting Metrology in Food and Nutrition

**DOI:** 10.3390/foods11040599

**Published:** 2022-02-19

**Authors:** Maria Z. Tsimidou, Stella A. Ordoudi, Fani Th. Mantzouridou, Nikolaos Nenadis, Tamara Stelzl, Michael Rychlik, Nastasia Belc, Claudia Zoani

**Affiliations:** 1 Laboratory of Food Chemistry and Technology, School of Chemistry, Aristotle University of Thessaloniki, 54124 Thessaloniki, Greece; steord@chem.auth.gr (S.A.O.); fmantz@chem.auth.gr (F.T.M.); niknen@chem.auth.gr (N.N.); 2 Chair of Analytical Food Chemistry, Technical University of Munich, 85354 Freising, Germany; tamara.stelzl@tum.de (T.S.); michael.rychlik@tum.de (M.R.); 3 National R&D Institute for Food Bioresources, IBA Bucharest, 021102 Bucharest, Romania; nastasia.belc@bioresurse.ro; 4 Italian National Agency for New Technologies, Energy and Sustainable Economic Development, Department for Sustainability of Production and Territorial Systems, Biotechnologies and Agro-Industry Division (SSPT-BIOAG), Casaccia Research Center, 00123 Rome, Italy; claudia.zoani@enea.it

**Keywords:** European research infrastructures, METROFOOD-RI, food metrology, agrifood, food authenticity, food safety, nutrition, One Health, reference materials

## Abstract

The pan-European distributed Research Infrastructure for Promoting Metrology in Food and Nutrition (METROFOOD-RI) has evolved in the frame of the European Strategy Forum on Research Infrastructures (ESFRI) to promote high-quality metrology services across the food chain. The METROFOOD-RI comprises physical facilities and electronic facilities. The former includes Reference Material plants and analytical laboratories (the ‘Metro’ side) and also experimental fields/farms, processing/storage plants and kitchen-labs (the ‘Food’ side). The RI is currently prepared to apply for receiving the European Research Infrastructure Consortium (ERIC) legal status and is organised to fulfil the requirements for operation at the national, European Union (EU) and international level. In this view, the METROFOOD-RI partners have recently reviewed the scientific plan and elaborated strategic priorities on key thematic areas of research in the food and nutrition domain to which they have expertise to contribute to meet global societal challenges and face unexpected emergencies. The present review summarises the methodology and main outcomes of the research study that helped to identify the key thematic areas from a metrological standpoint, to articulate critical and emerging issues and demands and to structure how the integrated facilities of the RI can operate in the first five years of operation as ERIC.

## 1. Research Infrastructures in Europe–METROFOOD-RI Evolution

Since 2002, the European Strategy Forum on Research Infrastructures (ESFRI) is the strategic instrument of the European Council (EC) “to support a coherent and strategy-led approach to policymaking on research infrastructures in Europe” [1]. As a result, facilities, resources, or services of a unique nature are identified by the European research communities as potent Research Infrastructures (RIs) to conduct and support top-level research activities in their domains. ESFRI selects proposals of strategic importance for the European Research Area (ERA), with excellent scientific case and an adequate level of maturity to become ESFRI Projects so that they can support their timely implementation as new or updates of RIs within a ten-year term. The successfully implemented RIs may gradually become ESFRI Landmarks. The latter are considered important elements of competitiveness of the ERA because they have proven capability of delivering science services and granting user access. RIs and Landmarks are expected to be optimally managed according to well-designed governance plans and legally supported by participating Member States [2]. Over the past decade, multilateral agreements for building up RIs appeared in all fields of science and technology, and several of them are already organised as European Research Infrastructure Consortia (ERIC) to fulfil the legal status requirements for their operation at national, European and international level.

The agrifood sector represents one of the largest and most important socio-economic sectors worldwide and also within the European Union (EU). The sector faces great challenges as practices prevailing for more than a century do not guarantee its resilience and fulfilment of the ultimate goal, i.e., “to feed the world”. Food security for all people at all times is currently challenged, not only because of global population growth, but also due to climate crisis, increasing poverty, and uncertainty that causes vast numbers of people to migrate at an uncontrolled pace. The consequences of the advancing COVID-19 pandemic have underscored the need for global cooperation in the sector. The serious disruption in the food chain that was experienced during the lockdown period had multiple negative effects, which are currently augmented by the energy crisis that hit primary production, manufacture, and trade of food products. The four pillars of food security, i.e., availability, access, utilisation, and stability were compromised worldwide, including Europe. Nevertheless, scientific and technological progress in the sector is continuous and all interested parties are eager to address current challenges in an effective and innovative way in accordance with global and EU initiatives and strategies. Priorities are set globally to promote sustainability of the sector with a focus on increasing agricultural yields and efficiency; limiting environmental burden on biodiversity, soils, water and air; reducing food losses and waste; and promoting patterns for healthier and less resource-intensive diets.

Following the deployment of the 17 Sustainable Development Goals (SDGs) by the United Nations General Assembly in 2015 (UN 2030 Agenda), many political initiatives at the global and EU level were put into force to implement the UN 2030 Agenda. Given that at least eight of the SDGs are directly or indirectly connected to agriculture, reliable and traceable measurements are crucial in supporting economic competitiveness, manufacturing and trade by ensuring traceability and sustainability of the agrifood systems in a circular economy prospect [3]. In this context, the pan-European METROFOOD-RI Infrastructure for Promoting Metrology in Food and Nutrition [4] was evolved to promote high-quality metrology services along the whole food chain, i.e., ’from farm to fork’, through FAIR (findable, accessible, interoperable, re-usable) data management practices. FAIR data have already been recognised as of strategic importance for the ERA [5].

**Figure 1:**
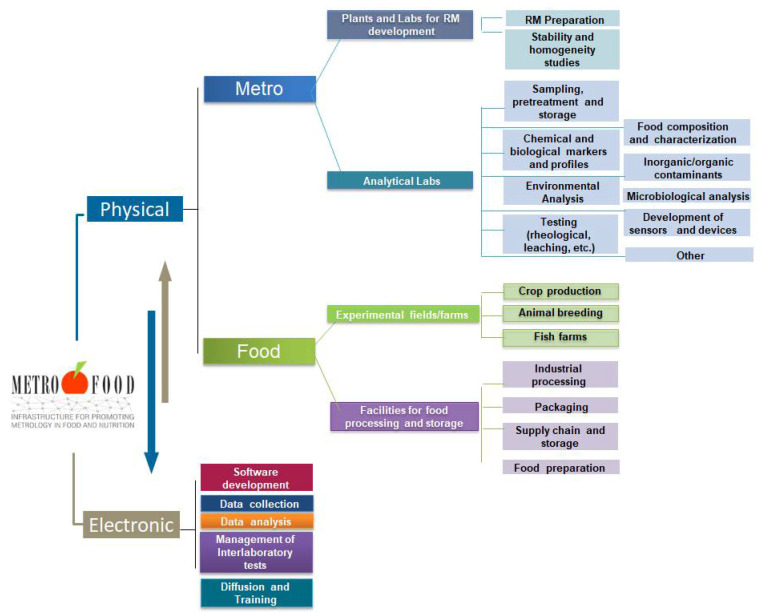
Basic service and operation components of the METROFOOD-RI. The Physical-RI consists of two sides, the ‘Metro’ and ‘Food’ ones that are integrated and coordinated by the Electronic-RI.

Currently, METROFOOD-RI runs its preparatory phase (PP), which is financed under the EU-Horizon 2020 project METROFOOD-PP (GA No 871083) to support its activities for self-assembly as a new distributed RI in the food and nutrition domain. It combines a physical and an electronic component with multidisciplinary facilities that are distributed across 18 European countries and can provide scientific services in an integrated and collaborative way (Figure 1). 

The physical component of the infrastructure has two sides: one consisting of Reference Material plants and analytical laboratories for the development and validation of new Reference Materials and new methods (the ‘Metro’ side), and another one consisting of experimental fields/farms for crop production/animal breeding, plants for food processing and storage, and kitchen-labs for food preparation (the ‘Food’ side). All physical facilities are integrated and coordinated by the electronic component of the infrastructure. The latter will provide an access platform to share and integrate knowledge and data on metrological tools for food analysis, focusing on food composition, nutritional value, safety issues, and authenticity markers. The METROFOOD-RI is well positioned in the ESFRI landscape and builds a strong network of facilities that are pertinent with many other domains, RIs and networks. Strengthening these links with joint strategies and complementary activities in a long-term schedule through the infrastructure operation requires a consolidated scientific plan of services in the domain. The development and continuous updating of a scientific plan is a core activity essential both for identifying strengths and weaknesses of the RI in relation to emerging topics of scientific research in food and nutrition, but also for aligning them with wider societal goals and challenges. The offer is addressed to a broad set of users, such as researchers and public and private laboratories; food business operators and producer associations; policy makers and food inspection and control agencies; and consumer associations and citizens [5]. This review covers the latest updating of the scientific plan of the METROFOOD-RI in view of its transition to full operation.

## 2. Scientific Priorities in the Food and Nutrition Domain from a Metrological Point of View

During its preparatory phase, the METROFOOD-RI prioritised some key thematic areas and identified demands for action in the agrifood sector. Thus, *The Future of Food and Agriculture: Trends and Challenges*, released in 2017 by the Food and Agriculture Organization (FAO), refers to the eradication of hunger and poverty by making agriculture and food systems more sustainable. Moreover, the EU Research and Innovation policy as declared in Food 2030 [6] focuses on four key food and nutrition goals including: (a) nutrition for sustainable and healthy diets; (b) climate-smart and environmentally sustainable food systems; (c) circularity and resource efficiency; (d) innovation and empowerment of communities. In this framework, the Food Challenge is also considered by ESFRI, to support Research Infrastructures that will address these challenges. As part of the strategic planning process, the One Health European Joint Programme (OHEJP) was launched, recognising that animal feed, human food, animal and human health, and environmental contamination are closely linked. Another integral part of the EC strategy for implementing the UN 2030 Agenda and the SDGs, with an overriding ambition of reducing net greenhouse gas emissions in the EU to zero by 2050, is the Green Deal. Its centrepiece comprises the so-called ‘Farm to Fork Strategy’, that has a strong focus on sustainable food production and processing; food security; sustainable food consumption and the shift to healthier and sustainable diets; food loss and waste; and food fraud.

In view of all the above, prioritisation of topics in the scientific context of the METROFOOD-RI became of utmost importance to enforce its strategic plan toward the provision of high-quality metrology services along the whole food value chain, through FAIR data management practices. 

To accomplish this, several EC policy documents were studied. A search on global industry initiatives and networks was also carried out to spotlight current demands and research trends. Above all, the latest scientific advancements in the broad field of metrology, covering not only food/feed matrices but also environmental matrices and food contact materials, were reviewed for the period 2015 onward. This particular search focused on scientific papers, review articles and books, as well as opinion papers that are accessible through the ‘Scopus’ and ‘Web of Science’ databases filtering out those with ‘PUBLICATION STAGE’ = final; ‘FIELD OF SCIENCE’ = Agricultural and Biological Sciences and ‘PUBLICATION YEAR’ = 2015–2020. The findings were classified into three wide thematic areas, as follows: (a) metrology in food, including Reference Materials and food integrity major components (food authenticity and traceability, food safety, food quality, nutritive quality and functional properties); (b) food security; and (c) sustainability of agrifood systems and circular economy. This review is focused on the first two thematic areas of the scientific plan, which are of great importance for the research and innovation agenda and the service development and delivery plan of the RI.

## 3. Key Thematic Areas in the METROFOOD-RI Scientific Plan

**Figure 2:**
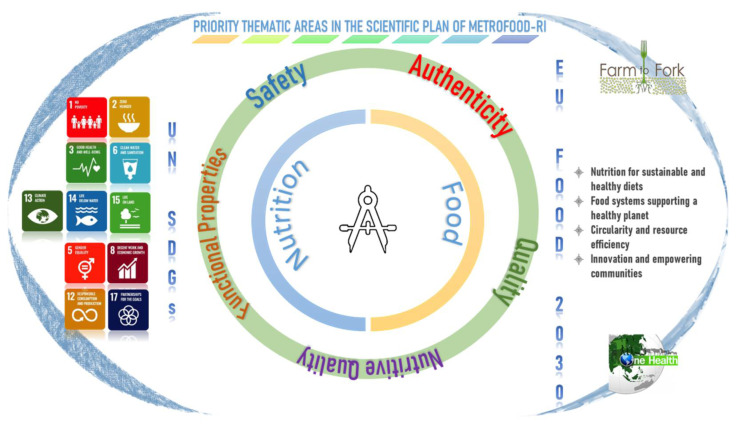
Key thematic areas covered in the METROFOOD-RI scientific plan and relevance to the global and EU policy framework in the agrifood sector.

The strategic priorities for Reference Materials and the major components of food integrity are illustrated in Figure 2 and discussed in the following subsections. General concepts, METROFOOD-RI capacity and perspectives are presented for each of the thematic areas.

### 3.1. Reference Materials 

The production of Reference Materials (RMs) is a key activity for the improvement and maintenance of a worldwide coherent measurement system. RM is a generic term that includes Certified Reference Materials (CRMs), matrix-RMs, primary or secondary measurement standards, calibrants (pure substances for calibration), and quality control materials [7]. METROFOOD-RI, aiming at supporting metrology in food and nutrition, provides as one of its core services the development and production of new RMs, which should be exploited to cover the aforementioned identified needs. 

CRMs are very important as they are also used to confirm or establish metrological traceability to conventional scales and determine the uncertainty of these results. CRMs play a key role in implementing the concept of traceability of measurement results in chemistry, biology, physics and other sciences dealing with substances and materials. For the production of (C)RMs, there is an International Standard and three ISO Guides that support manufacturing and certification to ensure that their quality complies with the requirements of the end users [8]. Matrix-RMs are of increasing popularity. In particular, the development of food-matrix (C)RMs has been associated with their increasing use by the analytical food testing community over the past 30 years. Even so, there is still a lack of fit-for-purpose (C)RMs in the agrifood sector. Several gaps exist, e.g., (C)RMs might not be available at all for certain matrices or matrix/analyte combinations, the range of parameters or available levels (concentrations) may not cover the analytical requirements, parameters needed might not be certified, and/or reference values might not be available for certain analytical processes (extractions, leaching, etc.). Thus, there is a continuous need to develop new RMs with different matrix/analyte combinations to cover existing and emerging analytical requirements [9]. This demand is related to many factors, such as the increasing innovation in analytical techniques, the development of new analytical methods suitable to detect/determine new parameters of emerging interest, the development of new profiling approaches (for quality, authenticity and traceability), and the need to support food safety and the accreditation of numerous laboratories, according to ISO/IEC 17025 requirements that led to greater demand for ensuring metrological traceability. 

#### 3.1.1. Capacity Building in Production

The available facilities of METROFOOD-RI for RM production, along with the further capacity expansion planned with upgrading, allow the development of new—even customised—matrix-(C)RMs covering new matrix/analyte combinations suitable to fulfil emerging analytical requirements (e.g., stable isotope analysis), as well as R&D activities on innovative RMs (e.g., multipurpose, single-use, double-phase, driven), and their production at both a small and a large (industrial) scale [10]. The most different matrix/analyte combinations can be considered, ranging from environmental matrices to food of animal/plant origin, beverages, extracts and essential oils, total diet and prepared foods, additives and supplements, wastes and by-products, non-food agricultural products, packaging and food contact materials, and so on. As for analytes, considered examples can be nutrients and health-promoting compounds, organic and inorganic contaminants, pesticide and veterinary drug residues, yeasts and moulds, metabolites, physical properties, genome sequences, etc. Regarding both matrices and analytes, priority should be given to emerging contaminants, mycotoxins, large molecular weight toxins, allergens, nanoparticles, microplastics, genetically modified organisms (GMOs) and DNA, sensory analysis and panel tests, authenticity and traceability of raw materials and products, by-products, and novel foods (insects included). The recently launched application RM-App as free e-service of the infrastructure (https://www.metrofood.eu/access/e-services.html) (accessed on 19 November 2021) is an open access tool supporting the search for ‘fit for the purpose’ RMs of interest specifically for the agrifood sector, covering the worldwide production of matrix-RMs and pure substances for calibration. Moreover, it helps to identify gaps for better planning and directing of future research activities of the infrastructure.

Table 1 presents some examples of emerging topics in this area that were prioritised in line with the key points of the Strategic Research Agenda for Metrology in Europe, defined by EURAMET [3], and suggestions made by the European Commission’s Joint Research Centre (JRC) [11].

Marketing needs for the declaration of geographical origin or a property in order to differentiate a product (e.g., health claims for olive oils—Polar phenols) can be the driving force for the development of RMs for identity or qualitative properties. The envisaged development of such RMs is expected to open new opportunities in the field of traceability and authenticity. At the same time, the metrological basis for setting the foundations of the estimation of uncertainty in the evaluation of nominal properties has already been laid down [16]. This approach will therefore allow the opportunity for producing RMs for sensory and texture analysis.

#### 3.1.2. Capacity Building in Characterisation

Characterisation is one of the steps in the production process of RMs that requires measurement of values for a variety of attributes and where calculation of uncertainties is necessary (e.g., for certified or reference values). RM characterisation requires minimally biased measurement procedures with low uncertainty as results will then be combined with those from homogeneity and stability assessment. Characterisation steps according to the recently revised ISO/Guide 35:2017 [17] can be achieved using (a) a single (primary) method in a single laboratory; (b) two or more independent reference methods in one or several laboratories; (c) one or more methods of demonstrable accuracy, performed by a network of competent laboratories; or (d) an approach providing method-specific, operationally defined property values, using a network of competent laboratories. It is evident that validated methods should be preferred when available. The involved laboratories should provide evidence of competence for the measurand in question independent of the measurements on the candidate CRM. Toward this direction, two further points should be taken into account. The first one is the point raised by the IAEA (International Atomic Energy Agency) regarding the inclusion of nuclear methods such as neutron activation analysis (NAA) for the characterisation of RMs when appropriate for the venture. This is due to the fact that these methods, although typically in the middle of the range with the three other major analytical methods (atomic absorption spectrometry, atomic emission spectrometry, mass spectrometry) with respect to precision, often exhibit superior accuracy. In addition, usually there is no need for sample preparation [11]. NAA performance is considered as equal to that of primary methods recognised by the Consultative Committee for Amount of Substance: Metrology in Chemistry and Biology (CCQM) [18]. In addition, it must be considered that NAA is one of the most suitable methods for performing homogeneity studies. The second point is the use of ID (isotope dilution)-LC-MS and ID-LC-MS/MS and also ID-GC-MS and ID-GC-MS/MS methods, which provide more accurately assigned values of lower expanded uncertainties, potentially < 3% depending on material homogeneity from a metrological point of view, to assign values for vitamins in supplements and foods instead of microbiological assays wherever applicable [13]. In the field of pathogens and GMO detection, digital PCR (dPCR) is recommended as a candidate reference method for the characterisation of DNA/RNA RMs, due to the precise quantification without the need for a reference standard [19]. Homogeneity and stability studies (under thermal and luminous stress) are also essential in order to properly develop RMs. 

#### 3.1.3. Perspective

The added value of METROFOOD-RI is that its physical infrastructure allows to combine the plants for RM development and production, with the whole spectrum of analytical laboratories that can be used for any type of chemical, physicochemical, and microbiological characterisation, even for the development of new multipurpose-Reference Materials, as well as for homogeneity studies. Furthermore, the ‘Food’ side physical facilities allow the in-house preparation of raw materials of well-known origin, which can be used to prepare new RMs, e.g., for authenticity studies (to validate geographical, botanical or zoological origin claims), or to develop driven-RMs, i.e., RMs naturally spiked directly in the field or in plants, thus much more representative of the naturally occurring contamination. As an example, METROFOOD-RI partners, together with other national and international institutes, recently participated in an inter-laboratory assessment (CCQM-K140) of stable carbon isotope ratio determination of bulk honey [20]. Finally, the integration with the e-component enables statistical analysis, data processing, organisation, and management of proficiency testing (PT). 

### 3.2. Food Authenticity and Traceability 

#### 3.2.1. Toward an Anti-Fraud Scientific Alliance 

Considering that food integrity is the state of being whole, entire, or undiminished or in perfect condition, the demand for accurate and standardised food authentication techniques is continuous [21]. Accumulating evidence for extended distribution of mislabelled or counterfeited products through the food supply chains triggers the need for objective criteria to protect conventional foods and products certified under various labels (regional, geographical indications, organic, etc.) in the EU. Food purity and authenticity is becoming an uprising issue for food authorities worldwide as it has been in the beginning of the 20th century because illegal practices can lead to severe health consequences for consumers and disrupt trust and fair trade. To update this part of the scientific plan of the METROFOOD-RI in view of the ERIC, policy views, initiatives, and research trends along with analytical breakthroughs on authenticity testing were taken into account. A literature search pointed out that important global and European initiatives to combat food fraud and sustain fair trade were established in recent years. Amendments to legislation in the U.S. and EU, Think Tanks and consumer fora for discussions and proposals, and collaboration between Europol and Interpol aim at combating the problem effectively. Special mention should be given to the AOAC International Standards and methods development program [22] that included actions on food authenticity methods covering targeted and non-targeted approaches in an effort to establish Standard Method Performance Requirements and adoption of single-laboratory or multi-laboratory validated methods. Such actions shape the metrology culture in the field of food authentication and highlight the importance of the complementary expertise existing among the METROFOOD-RI partners. The provision of high-quality analytical and metrological services for food authenticity testing will also require joint actions with other relevant networks in an open science environment. At a European level it is of utmost importance to connect with and support the activities of the recently established Knowledge Centre for Food Fraud and Quality (KCFFQ) [23] created by the EU Food Fraud Network and operated by the European Commission’s JRC. One of the main tasks of the KCFFQ is to create an expert network among the technical and scientific branches of Member States’ competent authorities dealing with food fraud for exchanging and disseminating food fraud related knowledge among the collaborating institutions and provide the necessary knowledge for evidence-based policymaking. The EU views on the currently available infrastructure for combating food fraud and identification of focus areas that need improvement were summarised and detailed in a recent communication [24], whereas implementation of this policy at the national level has been recently exemplified by Germany [25]. Participating EU Member State authorities have prioritised several issues for action against food fraud, some of which are fully aligned with the key objectives of the METROFOOD-RI from a scientific perspective. The creation of centres for analytical competence, harmonisation of analytical methods and creation of open access compositional databases for food commodities are among these top priority actions.

#### 3.2.2. State-of-the-Art Analytical Tools

A bibliometric search in the Web of Science database (accessed on 9 October 2021) using the keyword topic ‘food authentication’ signified that the field became extremely popular to scientific researchers since 2015, as the number of relevant publications has grown almost exponentially with regard to the previous decade. Thus, on average 300–500 research articles about novel analytical methods and food authentication techniques were published annually during this period. It is noteworthy that the number of review articles published in the abovementioned period about strengths and weaknesses of the applied protocols, food-matrix-related limitations and other relevant trends, and concepts and considerations was also very high (290 hits); 80% of these articles were published after 2018. The most cited articles in the field gather comprehensive information about data processing methods (e.g., chemometrics, multivariate modelling, data fusion) [26,27,28], blockchain technology for data storage [29], electronic sensors for in-line monitoring [30], non-targeted fingerprinting approaches [31], and DNA-based methods [32], and offer overviews of applications per food category [33] or analytical technology breakthroughs [34]. Despite the great wealth of information about the ‘proof of concept’ of the employed methodologies, the quality and comparability of the results that are critical for the transfer and application of the method into real food control systems are rarely discussed. The terms ‘metrology’, ’harmonisation’ or ’standardisation’ appear scarcely in the relevant publications. Some terms that are more specific with the evaluation of an analytical method performance such as ‘validation’ and ‘robustness’ are addressed in very few of those papers [28,31,35]. 

The major analytical tools are discussed below per wide categories of techniques among which chromatographic and spectroscopic ones were found to prevail.

*Chromatographic analysis*, which allows the resolution of complex mixtures into their constituents providing speciation and sensitivity, is, among others, a leading technology for food authentication. Traditionally, liquid (LC) and gas chromatography (GC) are the workhorses of official control laboratories [36]. Application for food authentication is challenging given that a great number of compounds including peptides, lipids, carbohydrates, amino acids, fatty acids, organic acids, nucleic acids, phytochemicals and other small molecule (additives, such as colorants, aromas, preservatives and other exogenous compounds) may be present in the test substrate. Authentication by chromatography is achieved after extraction and profiling specific groups of compounds such as fatty acids, triacylglycerols, sterols, hydrocarbons, tocopherols, and volatiles, which altogether form a characteristic profile (fingerprint) for food identity [34]. Matching the compound profiles with the pre-determined target values is the usual strategy of detection. During the last decade, high-resolution chromatographic techniques, such as gas (GC) or liquid chromatography (LC) coupled to mass spectrometry (MS), have emerged as powerful food authentication tools [34]. The analytical instruments used are Gas Chromatography Mass Spectrometry (GC-MS/MS), Liquid Chromatography Mass Spectrometry (LC-MS/MS) and Liquid Chromatography Time-of-Flight Mass Spectrometry (LC-TOF-MS) with tandem MS (triple quadrupole) gradually replacing older instruments in favour of non-targeted or targeted analyses, respectively. TOF or orbitrap analysers with electron spin (ESI) as the source of ionisation are proposed for non-targeted MS applications [37]. Ultrahigh pressure-operating HPLC (UHPLC) systems (up to 15000 psi) enable analysis with sub-2 micron columns in a significantly shorter time providing richer profiles for fingerprinting purposes [36]. UHPLC can be applied in tandem with mass spectrometry techniques to expand its potential. In addition, to overcome the target analytes overlapping, multidimensional chromatography, such as gas-gas (GCxGC), liquid-liquid chromatography (LCxLC) or hybrid systems (GCxLC), represents a suitable tool for analysis of complex food samples [38].

*Spectroscopic techniques*, based on the interaction of electromagnetic radiation with the matter that composes the food matrix, provide information about structural, physicochemical and compositional properties of the sample, through the wavelength or frequency detected in the emitted or absorbed energy spectrum [38]. Vibrational spectroscopy (mid-infrared (MIR), near-infrared (NIR), and Raman), ultraviolet–visible spectroscopy (UV–Vis), nuclear magnetic resonance (NMR), electron spin resonance (ESR), X-ray spectroscopy, and also mass spectrometry are widely used in the analytical applications. Among them, MIR, NIR, Raman and NMR spectroscopy are quite popular in food authentication studies. Vibrational spectroscopy techniques are versatile, non-destructive and non-invasive, offering indispensable advantages such as low cost, easy operation and low analysis time. Using vibrational spectroscopy allows, in some cases, the simultaneous detection of several compounds. NIR, MIR and Raman can be also employed in a complementary way; the combination (fusion) of multivariate data from various spectroscopic techniques is also gaining importance [26,35]. In recent years, the demand for hand-held, portable devices that allow in situ and high-throughput analyses for rapid, on-site food fraud inspection is on the rise [39]. Calibration of these devices using matrix-RMs and/or reference data from spectral libraries and databases is of utmost importance for ensuring a high level of performance in terms of sensitivity, specificity and reproducibility. Robust databases and transferable data from software to software are also needed. The fusion approach is expected to reduce the uncertainty of single-technique results, thus allowing for a better classification or prediction ability of the produced calibration models [35]. Molecular fluorescence spectroscopy is another simple, non-destructive and relatively low-cost analytical technique of high sensitivity. Aromatic bonds in polycyclic structures as well as heterocyclic compounds with rigid skeleton are some typical fluorophores of interest. Currently, simple, accurate and low-cost fluorimeters accompanied with advanced analytical software are available in the market giving, the opportunity for fast, reliable, reproducible measurements and spectra elaboration. Synchronous Fluorescence (SyF) that utilises concurrently scanned excitation–emission plots allows higher spectral selectivity resulting in stronger discrimination power and potential for food authentication application (e.g., olive oil adulteration) [34]. NMR spectroscopy represents an indispensable tool of analytical laboratories to elucidate molecular structures and to investigate the food composition at the molecular base. There is also an increasing interest in the development of applications for low-field benchtop instrumentation NMR that will allow its implementation in routine quality control for both targeted and non-targeted workflows [40].

NMR equipped with a deuterium probe and Isotope Ratio Mass Spectrometry (IRMS) that is principally measuring ratios of stable isotopes of light and heavy elements is considered a powerful tool for assessing geographical origin. Other technologies such as Multi Collector–Inductively Coupled Plasma–Mass Spectrometry (MC-ICP-MS), and Thermal Ionisation Mass Spectrometry (TIMS) or IR-MS interfaced with Elemental Analyser, Pyrolyser, Equilibration devices, GC or HPLC are used for the determination of light isotope ratios, while heavy isotopes are measured by MC-ICP-MS and TIMS [34]. The multi-isotope ratio analysis (2H/1H or D/H, 13C/12C, 18O/16O, 15N/14N, 34S/32S, 87Sr/86Sr) has found many applications to trace the geographical origin of wine, olive oil, orange fruit, honey, tomato, meat, dairy products, eggs, etc., or the growing system (organic vs. conventional). However, ensuring the validity of these measurements requires well-conceived and commodity-specific databases that are costly to compile and maintain. In recent years, the rare earth elements (REEs), including Y, Ce, Nd, Pr, Sm, Er, and Eu, have also been used in traceability studies. Many of the latter have demonstrated the usefulness of elemental profiling in tracing the origin of plant products. In the case of animal origin products, sources of variation related with animal feed have to be thoroughly studied in the future to strengthen the reliability of the employed methods [34,41]. It has to be stressed that the analysis of elemental isotopes for the identification of irradiated spices and even the detection of the level of irradiation [42] provides another traceability tool in the spice quality control. Recently, mass spectrometry has evolved as a stand-alone technique for elemental or molecular profiling and imaging, which are very sensitive approaches and require minimal sample preparation. Proton transfer reaction mass spectrometry (PTR-MS), Matrix-assisted laser desorption/ionisation Time-of-Flight Mass Spectrometry/MALDI-TOF-MS and Ambient Mass Spectrometry techniques such as Direct Analysis in Real Time/DART-MS or Liquid Extraction Surface Analysis/LESA-MS are some of the advanced MS variants of interest for food authentication studies. Quantitative on-line monitoring of volatile organic compounds (VOC) such as alkenes, alcohols, aldehydes, ketones, nitriles, sulphides and aromatic compounds allows sensitive determination in complex matrices. This potential was explored to uncover false description and mislabelling of high added value foods (e.g., saffron, truffle, honey, beer, olive oils, and juices), the botanical origin of spices, but also adulteration cases in olive oil, milk and coffee. The same applies for the quality control of animal feed [34]. 

*Molecular analysis* based on nucleotide- and protein-based methods is a well-established tool for the discrimination of fraudulent from authentic food products helping to identify false description or mislabelling. Among the DNA-based technologies of great potential for food authentication, DNA barcoding high-resolution melting (Bar-HRM), droplet digital PCR (ddPCR), high-resolution melting (HRM), loop-mediated isothermal amplification (LAMP), next-generation sequencing (NGS), polymerase chain reaction (PCR) and real-time quantitative PCR (qPCR), or also restriction-fragment-length polymorphism (RFLP), are popular methods [43]. Detection of GMOs, seafood authentication, authentication of kosher and halal meat certificates, detection of horse meat and pork in food labelled as beef, game meat authentication, botanical origin of foods (olive oil, wine, tomato products, tea, and cocoa), and species origin authentication (meat, milk, fish) are among the numerous useful applications [34]. Currently, next-generation sequencing technologies such as RNA-Seq are integrated with mass spectrometry-based proteomics to produce new analytical platforms that accelerate the pace of research at the interface of proteomics and genomics [44]. According to this proteogenomic approach, customised protein sequence databases that are generated using genomic and transcriptomic data help to identify novel peptides from the MS-based proteomic data; in turn, the proteomic data may provide protein-level evidence of gene expression and help to refine the gene models. The strength of this approach is that analysis becomes more specific and also scalable and allows for more systematic and comprehensive MS proteomic data mining. Most recently, advanced proteogenomic techniques such as PCR Single-Strand Conformation Polymorphisms (PCR-SSCP), random amplified polymorphic DNA (RAPD), or the emerging field of Peptide Nucleic Acid (PNA) and DNA fingerprinting have been applied in the field. Although the PCR-based molecular methods are selective, sensitive and repetitive, they are not an ideal option for forensic applications due to laborious and complex laboratory protocols, high costs, and infeasibility for on-site application [45]. Moreover, targeted detection and quantification of characterised molecular markers in real samples are still under way. This gap can be considered as an opportunity for capacity building within the METROFOOD-RI and a potential issue of priority in its scientific plan.

Among *immunological assays* with strong potential for food authentication, the enzyme-linked immunosorbent assay ELISA has been a leading option for several decades. Production of specific antibodies is a crucial step in the development of any immunoassay and further validation. So far, ELISA has been used to verify the authenticity of several food commodities such as meat, fish, and dairy products. It can also detect the presence of GMOs and undeclared processes such as food irradiation and can be considered as one of the classical tools in the armoury of METROFOOD-RI participating analytical laboratories.

An overview of the basic principles and instrumentation of the abovementioned techniques is given in Table 2.

#### 3.2.3. Perspective

Currently, there is an array of conventional and emerging analytical methods laying on different principles and instrumentation. Literature findings signify that there is still a lot of work to be completed before various analytical protocols become harmonised, legally robust or commercially relevant. To this direction, METROFOOD-RI will plan research and implement collaborative actions to promote metrology in food authentication and traceability applications. The consortium aims at providing consulting, training and analytical solutions to any interested parties (e.g., national authorities, industry).

It is generally accepted that food authentication workflows involve careful selection of representative and authentic reference samples, suitable analytical methods, reliable and unbiased chemometric analysis and unambiguous metabolite identification. The targeted approach, aiming at determining known molecules (e.g., the adulterant), is suggested when a suspect product or fraud incident needs to be confirmed and is very useful to support authenticity assurance to food manufacturers or to ensure the integrity of the food supply chain. The non-targeted approach requires the use of valid reference data sets and provides screening capability to safeguard that very few incidents may evade detection. Implementation of such methods into routine analysis and food surveillance requires thorough validation [37]. Consideration of current legislation gaps and communication with all interested parties is of utmost importance for the infrastructure in order for its scientific plan to be effective in this thematic area.

### 3.3. Food Safety 

#### 3.3.1. A matter of Societal Importance

Unsafe food can cause more than 200 different diseases, ranging from diarrhoea to cancers. Foodborne diseases can be both acute and chronic, and stem from biological, chemical, and physical sources. The legislative frame of the EU considers in a holistic way the safety of food, feed, animal well-being, plant protection and the environment in its General Food Law since 2000 when the European Food Safety Authority (EFSA) was established as the central authority to provide scientific support to the European Commission and the Parliament. Therefore, subsequent regulations, all types of documents, and current policies were the starting point for updating the safety component of the scientific plan of METROFOOD-RI from farm to fork. 

Starting from the farm and taking into consideration the recent report from the EC for statistics on pesticides, it is acknowledged that “pesticides are a cause of pollution and have a direct effect especially on the state of biodiversity, water bodies, and soils. To ensure that these impacts are addressed appropriately, it is essential that policy makers are able to quantify the risk and the level of pesticide pollution” [46]. Currently, the policies concerned by the data needs are the EU Biodiversity Strategy 2020, the Common Agricultural Policy (CAP), the Water Framework Directive, and the Thematic Strategy on Soils. The CAP 2020 will add two important directives in the conditionality for farmer’s payments—the Water Framework Directive and the Directive on the Sustainable Use of Pesticides—so it is extremely important to identify the gaps and propose ways to orient the METROFOOD-RI facilities toward emerging risks and legal requirements. According to relevant EU reports concerning the sustainable use of pesticides [47], it is acknowledged that, “The EU framework aims to achieve sustainable use of plant protection products (PPPs) by reducing risks and impacts on human health and environment and promoting integrated pest management”. Both the EU Commission and Member States have taken action to promote the sustainable use of PPPs, but there has been limited progress in measuring and reducing the associated risks. Consequently, it is mandatory to develop eco-sustainable alternative tools that might be of inclusion into the low risk [48] or basic substances (Article 23) catalogues. 

In the following paragraphs, major sources of hazards along with current analytical gaps and challenges that METROFOOD-RI scientific plan prioritises are discussed.

#### 3.3.2. Capacity Building in Assessing Chemical Hazards

Chemicals are the biggest group of potential hazards in the food chain, because, in general, all of them may be toxic. Chemicals can be found in food either intentionally, when used for a technological reason (e.g., food additives), or due to air, water and soil pollution. Chemicals in food are considered globally as a top safety issue and are of concern in international trade transactions [49]. Contaminants [50] are substances that are not intentionally present in food. They may be found in foods as a consequence of the various steps of its production, packaging, transport and storage. Additionally, new toxic residues in food are detected due to the application of new industrial processes, agricultural activities, and environmental pollution. The so far identified chemical contaminants include structurally a wide range of compounds. They differ in size, stability, functional moieties, and toxicity. Their determination is usually challenging for the analysts. The late reports of Rapid Alert Food and Feed portal (2017, 2018) [51,52] show the prevalence of mycotoxins, pesticide residues, allergens, metals, environmental pollutants, and process contaminants as serious or serious with undecided risk contaminants.

Contaminants of emerging concern or emerging contaminants are compounds with either no defined maximum levels in the EU legislation yet, or having maximum levels, which need to be revised [53] because of new data availability or the development of new analytical tools. A literature search for the 2015-onward timespan indicates the prevalence of perfluorinated compounds (PFCs), polybrominated biphenyls (PBBs), nanomaterials, marine biotoxins such as palitoxins and spirolides, and the new generation of pesticides, antibiotics and coccidiostats. Emerging mycotoxins (enniatins, beauvericin, moniliformin, fusaproliferin, fusaric acid, culmorin, utanolide, sterigmatocystin, emodin, mycophenolic acid, alternariol, alternariol monomethyl ether, and tenuazonic acid) have also been recently proved to be present in agrifoods and feeds, exerting serious toxicity effects. Antibiotics, persistent organic pollutants (POPs) namely, PBDEs, PCBs, OCs, PAHs, and OPs, perfluoroalkyl substances (PFASs) and parabens (endocrine disruptors) are of increasing concern. Lately, there is growing concern about the presence of microplastics in the environment and their subsequent transfer in the food chain [54,55]. 

Lack of methods of analysis or methods with poor performance that are in use do not facilitate the progress for their effective control in foods. In the field of instrumental methods, the chromatographic ones coupled to different MS detectors (e.g., LC-MS/MS, LC-QTOF-MS, GC-MS) is still the most suitable means to detect contaminants in foods. There is a trend in employing both targeted and non-targeted high-resolution-MS methods (HRMS), which detect a wider array of compounds (multiclass), such as the one developed by Steiner et al. [56] for the detection of more than 1200 biotoxins, pesticides and veterinary drugs in a complex feed and that of Rausch et al. which detects 40 mycotoxins, 2 plant growth regulators, 2 tropane alkaloids, and 334 pesticides in cereals [57]. TOF accurate mass techniques can be applied for the non-targeted identification of pesticides, their metabolites, or degradation products and other unknown compounds present in the samples [58]. These HRMS methods, together with large volume solid phase extraction and passive sampling are recommendations made by NORMAN, an independent organisation at the interface between science and policy [58], as these approaches enable the detection of traces of emerging contaminants. The necessity for confirmatory analysis as an important aspect in food chain sustainability is also a must, although the cumbersome sample preparation is still a challenge that has to be resolved somehow in the future. Another challenge, in the frame of green approaches, is to reduce the solvent consumption during the course of analysis. More attention should be given to screening methods in order to reduce the population of samples to be analysed by separation methods or to promote the applicability of non-invasive methods of analysis. Smartphones can reform the present food testing status [59] by enabling farmers or consumers to examine the foods by themselves. Nonetheless, validation and benchmarking issues have to be considered carefully to verify method reliability, limit false-negative results, and that they are fit for the purpose. 

#### 3.3.3. Capacity Building in Assessing Biological Hazards

Sources of biological hazards include bacteria, viruses, parasites, and prions, some of which have caused serious damage to human health and economic losses. Foodborne bacteria such as *Salmonella spp*. (e.g., *S. enterica*), *Listeria monocytogenes*, *Escherichia coli* O157 and other Shiga-toxin producing *E. coli* strains, *Staphylococcus aureus* strains coding for thermostable enterotoxins, *Campylobacter* spp., *Clostridium* spp., *Bacillus cereus* and *Vibrio* spp., viruses (Hepatitis A and Noroviruses), parasites *(Cyclospora cayetanensis, Toxoplasma gondii* and *Trichinella spiralis)* and mis-shaped prions (PrPres), are leading causes of foodborne diseases. In addition to human health risks, microbial contamination, which is assigned as a new hazard category that comes from ‘non-pathogenic microorganisms’, such as *E. coli* or *Enterobacteriaceae*, can result in food spoilage [60,61,62,63,64]. 

In line with the global *One Health* approach and in order to control/prevent human exposure to biological hazards through food, the European Commission has set up a comprehensive legal framework based on the scientific advice from EFSA to improve food safety in Europe. The ultimate goal is “a co-ordinated and holistic approach towards food hygiene, covering all levels of the food chain, applying a transparent hygiene policy to all food operators and ensuring an efficient, risk-based and independent control” [64]. The efforts are focused on enhancing knowledge of pathogen origins and trends by monitoring zoonotic agents across the food and animal feed chain. Programs are developed to control *Salmonella* and other foodborne zoonotic disease and to reduce the risk to public health. Emphasis was also given to the establishment of microbiological criteria that can be applied both at the different stages of food production and to food items already on the market, and on harmonisation of control measures against transmissible spongiform encephalopathies (TSE, BSE, scrapie) to prevent contagion of other animals or consumer exposure [64].

*Salmonella* spp. and *Listeria monocytogenes*, which are the most frequently reported pathogens in food from EU Member States, are mostly found in foods of animal origin (e.g., poultry and poultry products, other meat and meat products, milk and milk products). In non-EU Member States, pathogenic microorganisms are among the top issues for products. Focusing on the high-risk pathogen *Salmonella*, recent outbreaks, however, have increasingly been tied to seeds and vegetables [52]. The latter statement is also supported by the INFOSAN activity report 2016/2017 and frequent incidents in the USA and elsewhere that led the Food and Drug Administration (FDA) to announce the New Era of Smarter Food Safety from July 2020 [65]. According to the latter document, “achievable goals to enhance traceability, improve predictive analytics, respond more rapidly to outbreaks, address new business models, reduce contamination of food, and foster the development of stronger food safety cultures” are reported. Obviously, the COVID-19 pandemic consequences in the USA fostered the need for these strategic initiatives. 

Existing reference methods in food microbiology are mainly conventional *culture-based methods* that are considered to be simple, inexpensive and sensitive, though laborious and with important metrological constraint (false-negative results). Despite considerable analytical improvements and innovative revisions over the years, culture-based methods are still considered as conventional. Frequently, conventional culture methods may be proved a gold standard so that METROFOOD-RI pays attention both to these means as well as to alternative methods [65] for pre-processing and rapid/direct, target-specific culture-independent detection of foodborne pathogens in food samples, even at low level. The latter are on the rise as they are characterised by high sensitivity, specificity, accuracy, and reproducibility, and also suitability for in situ analysis. These methods provide efficient separation and concentration techniques of the target pathogens (antibody-based, physical- and chemical-based techniques), which are necessary in further analysis using nucleic-acid-based methods (simple polymerase chain reaction (PCR), multiplex PCR, real-time PCR, nucleic acid sequence-based amplification (NASBA), loop-mediated isothermal amplification (LAMP), and oligonucleotide DNA microarray), immunological methods (enzyme-linked immunosorbent assay (ELISA) and lateral flow immunoassay), and biosensor-based methods (optical, electrochemical, and mass-based biosensors) [66,67].

*Molecular typing* by applying PCR techniques and sequencing has been developed rapidly worldwide. Data on molecular typing of foodborne pathogens can contribute strongly to surveillance and outbreak detection [68]. According to the EFSA opinion of 2019 for Whole Genome Sequencing (WGS) [69], “WGS offers the highest level of bacterial strain discrimination for foodborne outbreak investigation and source-attribution as well as potential for more precise hazard identification, thereby facilitating more targeted risk assessment and risk management. WGS improves linking of sporadic cases associated with different food products and geographical regions to a point source outbreak and can facilitate epidemiological investigations, allowing also the use of previously sequenced genomes”.

Due to the nature of the analyte (i.e., living microorganism) and the different principles of the various methods used in food microbiology, harmonisation of methods through standardisation is of high importance to ensure comparability of results and to allow data exchange. Comprehensive testing and validation of alternative methods by collaborative studies will support the future development of a new generation of reliable foodborne pathogen detection technologies [69]. For example, in the abovementioned EFSA opinion, it is recommended that “…international organisations for standardisation provide guidelines covering the entire process from DNA extraction to final result. In addition, further harmonisation and transparency in relation to the bioinformatic approaches, reference sequences and software developments for the analysis of WGS and metagenomics data are required. Capacity building for WGS (and metagenomics) within European laboratories and also worldwide is important to increase information exchange and associated benefits” [69]. In this regard, a great number of EU reference laboratories (EURL) and national reference laboratories (NRL) networks work on the field of biological food safety, and more specifically on molecular testing. Several EURLs regularly organise typing training sessions and proficiency testing trials with the aim to consolidate typing capacity of the NRL network [70]. 

The multi-omics approach integrating proteomics, metabolomics, metagenomics, and transcriptomics has also been proposed for the development of analytical methods to detect food pathogens rapidly in food and also to define potential biomarkers. In this direction, validation of biomarkers should be considered as the next most important stage within METROFOOD-RI capacity building for addressing microbial safety [71]. 

#### 3.3.4. Perspective

Establishing competence of METROFOOD-RI in this area seems to be a priority given that each contaminant has to be determined in a variety of food products. METROFOOD-RI experts need to collaborate in proficiency testing in order to validate matrix-specific methods. Benchmarking toward instrumental methods and the emergence of smartphone-based methods for the detection of food contaminants should be one of the priorities among experts in the RI40 technology. Automation and IT tools from the e-component of the RI can be combined with the facilities of the Physical-RI to reduce time and cost and favour simplicity. In addition to the existing capability for standardisation of data generation and data analysis, upgrading e-platforms for data sharing will be also valuable in the direction of global control of food hazards (e.g., foodborne pathogens [72]).

### 3.4. Food Quality 

#### 3.4.1. What and How We ‘Measure’ It

The concept of ‘food quality’ encompasses numerous intrinsic and extrinsic characteristics of a food (raw materials, semi-processed/processed products) and all activities/services that take place from farm to fork. All interested parties (scientists, industry, authorities, consumers) influence decisions on the number of key quality attributes for each food product/commodity that should be considered at a given time period. Then, these attributes are either covered by legislation or become part of the internal specification armoury of each production unit. Quality control and quality management systems play a crucial role in the establishment of a product in the market. Quality standards in the form of trade standards can be public or private, open access, or by payment (membership fee, or standard purchase). Globalisation encouraged uniformity of quality food standards, harmonised units, serving sizes, labelling, etc. However, the continuous changes in the agrifood sector and the substantial diversity in food policies in the developed countries and emerging economies (GMOs, hormones, country of origin, religious constraints, novel foods, new technologies, different diet patterns, bio-security, electronic trade, etc.) have created a complex, not always fair environment for food commerce. A product of ‘excellent’ quality should fulfil all specifications and consumer expectations including ‘extra’ benefits in comparison to any other product of its kind that can be found in the market. Any judgment about the degree of compliance of a product with a standard should be in a ‘non-arbitrary’ and impartial way so that it is of utmost importance to ’quantify’ quality attributes in an objective manner.

#### 3.4.2. Perspective

The analytical labs of METROFOOD-RI cover well the feasibility of relevant physicochemical, microbiological/biological and organoleptic analysis of foods. In addition, following metrological concepts including, among others, high-quality reference standards, validated methods, robust sampling practices, proven calibration approaches, natural matrix-reference materials, speciation chemistry, assessment of measurement uncertainty and proficiency testing, the consortium may provide reliable data that can solve problems facilitating national/regional development, trade, and public health decisions, and contribute to nutrition education.

Τhis part of the scientific plan encompasses the emerging trend of using modern high-throughput non-destructive instruments, often called ’food scanners’ such as those based on spectroscopic methods. These devices are expected to facilitate numerous food sample testing procedures, thanks to minute sample amount and minimal or no sample pre-treatment, the availability of advanced chemometrics data handling tools, wireless data communication and ‘Big Data’ compatibility. Smartphone applications will assist near-future expectations such as the ability of food inspectors, farmers, retailers and consumers to test the quality characteristics of foods on their own [73]. On the other hand, processing facilities and kitchen-labs of the infrastructure supported by sensory panels have the expertise to contribute to the quality control and acceptability of reformulated or new products. Sensory analysis approaches that rely on well-trained panellists, even when standards are available, may introduce uncertainty issues because individuals exhibit different sensitivities, preferences, and product knowledge. The development of instrumental techniques that could recognise objectively and quickly specific sensory characteristics in the same way as an expert tasting panel perceive them is, thus, in high demand. Except for Gas Chromatography Olfactometry (GCO), biomimetic sensors such as electronic tongue (e-tongue), electronic nose, (e-nose), electronic eye, (e-eye) and computer vision systems (CVSs) are some of the available tools [30] that METROFOOD examines from the metrological point of view. 

### 3.5. Nutritive Quality and Functional Properties

#### 3.5.1. Nutrients and Beyond

Food–health relationships are well established for the average consumer of the 21st century, who receives multiple types of information through official documents (regulations, EFSA portal, local authorities, dietary guidelines) and unofficially through electronic and conventional sources for positive and negative attributes of food consumption. Consumer information and education is a priority for the EU Member States to avoid adverse health effects mainly through awareness and prolepsis. The current trend in nutrition is toward foods tailored to the individual (personalised/precision nutrition) and aimed at a healthier and effective diet. At the same time, a strong demand for package-free and local food products, a decline in the consumption of animal-based foods and a preference in health-promoting and functional food ingredients can be observed [74]. Advances in food science and technology have contributed to the provision of a wide variety of foods that are safe, convenient, affordable, and widely available throughout the year [75]. At the same time, population growth, climate change accompanied by related harvest losses, globalisation but also abrupt disruption of the food chain, raise new concerns and set priorities that require global synergies up to 2030 and onwards. In the light of a worldwide change in human consumption habits, accompanied by an increase in diet-related diseases primarily in industrialised nations—while hunger and malnutrition are rampant in most developing countries—the aspects of quality and nutritive value of food have become of central importance in nutrition research. Food wholesomeness is determined by the quantity and quality of nutrients contained therein and their harmlessness for human health [76]. Fresh food is widely purchased by consumers who can afford it as it is considered a healthier choice. Particularly for processed foods, nutrition-conscious consumers are paying increasing attention to the ingredients and are demanding high-value and healthy foods, even though the interpretation and definition of the term ‘healthy food’ is not consistently agreed upon experts [77]. Functional foods are a promising segment of the agri-business, even if this term still lacks a universally accepted definition for these products. Generally, they are defined as foods offering additional benefits that may reduce the risk of disease or promote optimal health when they are part of an everyday diet. In the face of increased health care costs related also to a higher occurrence of pathologies correlated with poor eating habits, clinical and epidemiological studies show that a healthy and balanced diet, rich in fruits, vegetables, whole grains, fish, and dairy products, and low in saturated fat and sodium, brings numerous benefits and can reduce the risk of diseases such as cardiovascular disease, hypertension and some types of cancer [77]. The introduction and exploitation of functional foods represents a great opportunity for the re-evaluation of traditional food products as well as the development of new products rich in bioactive substances. In order to ensure that any claim made on a food label is clear and substantiated by scientific evidence, European authorities issued the EC Regulation 1924/2006 concerning the use of nutrition and health claims. EFSA must verify the scientific substantiation of the petitions that have to fulfil all requirements according to specific guidelines [78]. 

#### 3.5.2. State-of-the-Art Analytical Tools

As the determinants of general nutritive quality, proximates such as lipids, carbohydrates, proteins, and minerals have to be considered. For these, the golden standards are general reference methods collected under the ISO system. These are essential for defining, e.g., the nutritive energy of foods. Moreover, within lipids and proteins, the content of essential components such as essential amino acids and essential fatty acids has to be determined by amino acid analysis and high-resolution gas chromatography [79]. Amino acid profiling will also define basic protein quality. Regarding the complex group of carbohydrates, the content of soluble and in-soluble fibre [80] is important for defining the beneficial impact on gastrointestinal health.

For trace nutrients such as elements and vitamins, the methodology is much less standardised and under on-going development. As trace quantitation is more susceptible to analytical errors and statistical imprecision, the methods require increased sensitivity and specificity, which is effectively achieved by mass spectrometric methods. However, although mass spectroscopy is highly specific and sensitive, signal intensity is often impeded by matrix effects. Moreover, extensive sample preparation often includes inferior recoveries, which require suitable compensation by internal standards. For this purpose, stable isotopologues are the compounds of choice and the respective methods are termed stable isotope dilution assays (SIDA). The most critical bottleneck for using SIDAs is the availability of the stable isotope labelled standards, and, therefore, new SIDAs are mainly elaborated by scientists that are experienced in synthesising these compounds. For vitamins, SIDAs based on LC-MS/MS detection have been reported for the B6 group [81], the folates [82,83], and vitamin B12 [84]. For elements, SIDAs are mainly based on ICP-MS detection.

For the quantitation of health-promoting compounds and bioactivities, the method portfolio is even more diverse and less standardised. For example, the vast group of phenolic compounds can be quantified by LC-MS/MS methods or by GC-MS after derivatisation, but due to the large number of compounds, SIDAs are rare. For evaluating health-promoting activity, in vitro assays for antioxidant capacity likely have the longest history. However, different protocols such as the 1,1-diphenyl- 2-picrylhydrazyl radical (DPPH radical) assay, oxygen radical absorbance capacity (ORAC) or the ferric reducing antioxidant power (FRAP) assay are in use and their results are not comparable and often conflicting [85]. Other bioactivity assays require the use of cell cultures, and therefore the results are even more so only indicative. Examples for these types of bioactivities are antimicrobial, anti-inflammatory, and antihypertensive properties. Here, the demand for standardisation is even higher to make the results comparable, reproducible, and includable in databases.

#### 3.5.3. Perspective

According to expert perceptions, METROFOOD-RI should address in its scientific plan the following topics as summarised in Table 3.

For all these goals, accurate analytical methods for assessing the nutritive quality and health-promoting properties are essential.

The current methodological state of the art and the method portfolio available at METROFOOD-RI ensures the availability of laboratories quantifying carbohydrates, triacylglycerols/fatty acids and proteins/amino acids, as well as vitamins and minerals. Therefore, there is broad expertise within the consortium to assess the nutritive quality of foods and, by combining these labs with the facilities for RM production, to develop and produce new RMs fit for these analytical purposes. In the food production sector, METROFOOD-RI is highly specialised in food processing (e.g., development of pilot plants or innovative and mild food processing technologies), primary production (e.g., plants, livestock, seeds, feedstuffs), and food packaging technology (e.g., smart and active packaging solutions). With regard to nutritive quality, METROFOOD-RI already includes many analytical laboratories commonly applying general reference methods for proximates. 

## 4. Conclusions

The overview of the latest scientific advancements in the wide key thematic areas where the METROFOOD-RI is active helps to highlight gaps and research trends in metrological issues related to food and nutrition that are of particular interest for upgrading the future services of the RI in the health and food domain. Summarising the research outcomes and views on each topic, it became possible to identify sub-targets of action. Horizontal activities aiming at increasing analytical testing competences, harmonising research methodologies, sharing of experience, best practices and networking, and fostering cross-border knowledge transfer through access to data/databases and analytical methods seem to be fundamental. Future goals of the METROFOOD-RI scientific plan include development of reliable and traceable diagnostic systems (methods and devices) and Reference Materials for quality, safety and traceability of raw materials and products; development of authenticity and traceability markers using targeted and non-targeted approaches; study of multiple exposure to different chemicals; emerging pesticides and mycotoxins; feed safety; microplastics; examination of effects of innovative technologies (e.g., nanotechnologies) to food quality and safety, and examination of the nutritive quality and wholesomeness of food with emphasis on nutrients and other functional constituents. METROFOOD-RI distributed facilities can face the current challenges of the agrifood sector and play a leading role in bringing together fragmented capabilities to form an integrated unit of excellence in the thematic areas articulated throughout the text.

## Figures and Tables

**Table 1:** Identified gaps in the field of Reference Material (RM) design and production and emerging topics that are of high priority for the METROFOOD-RI consortium.

Reference	Identified Gaps and Emerging Topics
[12]	traceable measurements of residues and pathogens (viruses, bacteria, toxins)
	RMs for species identification, embedded nanoparticles microplastics, allergens
[13]	additional (C)RMs in the less-populated sectors of the protein-fat-carbohydrate (P/F/C) AOAC (Association of Official Analytical Collaboration, International) triangle
[14]	RMs with assigned values for vitamin D and its metabolites, vitamin K and folate vitamins
	RMs for arsenic parameters, emerging contaminants, persistent organic pollutants in marine biota, GMOs, alkaloids, mineral oil hydrocarbons, glyphosate in cereals (for which there is a controversy on Maximum Residue Level), food contact materials migration, cocoa
[14,15]	RMs for acrylamide in other than infant formula products
[16]	RMs for sensory analyses, panel tests, authenticity (markers/profiles), identity or other qualitative properties

**Table 2:** Overview of analytical techniques commonly used in authenticity and traceability studies [34,35,36,37,38,39,40,41,42,43,44,45].

Analytical Technique	Principle		Variation
*Chromatographic*			
(Ultra) High performance liquid chromatography (U)HPLC	Adsorption and/or partition of target analytes between mobile (liquid or gas) and stationary phase	Separation	Hyphenation to spectrometry MS/MS TOF Triple quadruple
Gas chromatography (GC)			
Multidimensional chromatography (LC x LC, GC x GC, GC x LC, LC x GC)			
*Spectroscopic*			
Infrared	Absorption of electromagnetic radiation	Vibration of bonds of molecular functional groups (prerequisite change of dipole moment)	Fourier-Transform-Mid-infrared (FT-MIR) Fourier-Transform-Near-infrared (FT-NIR)
Raman		Vibration of bonds of molecular functional groups (prerequisite change of polarizability)	Raman Fourier-Transform-Raman (FT-Raman)
Ultraviolet–visible (UV–Vis)		Excitation of electrons	
Fluorescence		Energy emission after atom excitation to higher energy levels	Synchronous (SyF) Front phase (FP)
Nuclear magnetic resonance (NMR)	Absorption of radiofrequency radiation by atomic nuclei with non-zero spins	Resonance	High-resolution NMR Low-resolution NMR Liquid/solid-state
X-ray	Absorption and scattering of X-ray beams	Image	X-ray fluorescence (XRF)
Mass spectrometry	Formation of ions with different mass-to-charge ratio	Separation in an electromagnetic field	Isotope Ratio (IR-MS), Inductively Coupled Plasma (ICP-MS) Thermal Ionisation (TI-MS) Proton transfer reaction (PTR-MS) Matrix-assisted laser desorption/ionisation Time-of-Flight (MALDI-TOF) Direct Analysis in Real Time (DART) Liquid Extraction Surface Analysis (LESA)
*Molecular*			
Polymerase chain reaction (PCR)	Amplification of DNA fragments	Separation of DNA fragment sizes by gel-electrophoresis (sequencing), melt curves of DNA fragments	DNA barcoding high-resolution melting (Bar-HRM) Droplet digital PCR (ddPCR), High-resolution melting (HRM), Loop-mediated isothermal amplification (LAMP), Next-generation sequencing (NGS) Polymerase chain reaction (PCR) Real-time quantitative PCR (qPCR), Restriction-fragment-length polymorphism (RFLP) PCR Single-Strand Conformation Polymorphisms (PCR-SSCP) Random amplified polymorphic DNA (RAPD), Peptide Nucleic Acid (PNA) DNA fingerprinting
*Immunological*			
Ligand binding (LB)	Complex formation between antigen (target protein) and antibody	Production of a detectable signal (usually colour)	Enzyme-linked immunosorbent assay (ELISA)

**Table 3:** High-priority topics in the scientific plan for nutritive quality and functional properties.

	Topic
1	Exploration of the health benefits of nutrients with enhanced value and structural function, such as phytonutrients.
2	Development of new technologies need to be developed for fractionation, isolation, extraction, reformulation (e.g., low salt content), concentration and delivery of health-promoting ingredients.
3	Raising the volume of food, feed, and fibre by reducing waste from food processing and by-product recovery. Development of novel processes for production of food products with high-value components (superfoods) and development of new processing technologies to protect and concentrate nutrients such as phytonutrients, vitamins, and flavour/aroma phenols.
4	Development and implementation of methods to improve processing and end-product quality and rapid measurement techniques for functionality and nutrient prediction.
5	Development of healthy, flavourful, and value-added food products to both maximise health effects and combat nutrition-related diseases.
6	Research on new administration techniques for nutrients (e.g., probiotics, nano-emulsions) and development of new processing technologies for the identification, characterisation, stabilisation, and delivery of nutrients
7	Development of knowledge and insight into the interaction between bio-metabolism and nutrients/food interactions.
8	Fostering quality improvements through research on foods and feed with increased added value, improving the quality of harvested and processed products, the quality of products in controlled atmospheres and reducing quality losses during storage. Establishing post-harvest practices toward optimising food quality through enhanced monitoring.
